# SARS-CoV-2 presence in recreational seawater and evaluation of intestine permeability: experimental evidence of low impact on public health

**DOI:** 10.3389/fpubh.2024.1326453

**Published:** 2024-03-04

**Authors:** Clelia Norese, Elena Nicosia, Katia Cortese, Valentina Gentili, Roberta Rizzo, Sabrina Rizzo, Elena Grasselli, Giulia De Negri Atanasio, Maria Cristina Gagliani, Micaela Tiso, Matteo Zinni, Alessandra Pulliero, Alberto Izzotti

**Affiliations:** ^1^DIMES, Department of Experimental Medicine, University of Genoa, Genoa, Italy; ^2^Regione Liguria, Environmental Department, Ligurian Region, Genoa, Italy; ^3^Department of Chemical, Pharmaceutical and Agricultural Sciences, University of Ferrara, Ferrara, Italy; ^4^LTTA, Clinical Research Center, University of Ferrara, Ferrara, Italy; ^5^Department of Earth, Environmental, and Life Sciences (DISTAV), University of Genoa, Genoa, Italy; ^6^MICAMO, Spin-Off Department of Earth Sciences, University of Genoa, Genoa, Italy; ^7^DISSAL, Department of Health Sciences, University of Genoa, Genoa, Italy; ^8^HSM, IRCCS Ospedale Policlinico San Martino, Genoa, Italy

**Keywords:** SARS-CoV-2, COVID-19, seawater, saltwater, infectivity, public health

## Abstract

**Introduction:**

Coastal seawater pollution poses a public health risk due to the potential ingestion of contaminated water during recreational activities. Wastewater-based epidemiology has revealed the abundant presence of SARS-CoV-2 in seawater emitted from wastewater outlets. The objective of this research was to investigate the impact of seawater on SARS-CoV-2 infectivity to assess the safety of recreational activities in seawater.

**Methods:**

Wild SARS-CoV-2 was collected from oral swabs of COVID-19 affected patients and incubated for up to 90 min using the following solutions: (a) standard physiological solution (control), (b) reconstructed seawater (3.5% NaCl), and (c) authentic seawater (3.8%). Samples were then exposed to two different host systems: (a) Vero E6 cells expressing the ACE2 SARS-CoV-2 receptor and (b) 3D multi-tissue organoids reconstructing the human intestine. The presence of intracellular virus inside the host systems was determined using plaque assay, quantitative real-time PCR (qPCR), and transmission electron microscopy.

**Results:**

Ultrastructural examination of Vero E6 cells revealed the presence of virus particles at the cell surface and in replicative compartments inside cells treated with seawater and/or reconstituted water only for samples incubated up to 2 min. After a 90-min incubation, the presence of the virus and its infectivity in Vero E6 cells was reduced by 90%. Ultrastructural analysis performed in 3D epi-intestinal tissue did not reveal intact viral particles or infection signs, despite the presence of viral nucleic acid detected by qPCR. Indeed, viral genes (Orf1ab and N) were found in the intestinal luminal epithelium but not in the enteric capillaries. These findings suggest that the intestinal tissue is not a preferential entry site for SARS-CoV-2 in the human body. Additionally, the presence of hypertonic saline solution did not increase the susceptibility of the intestinal epithelium to virus penetration; rather, it neutralized its infectivity.

**Conclusion:**

Our results indicate that engaging in recreational activities in a seawater environment does not pose a significant risk for COVID-19 infection, despite the possible presence of viral nucleic acid deriving from degraded and fragmented viruses.

## Graphical abstract

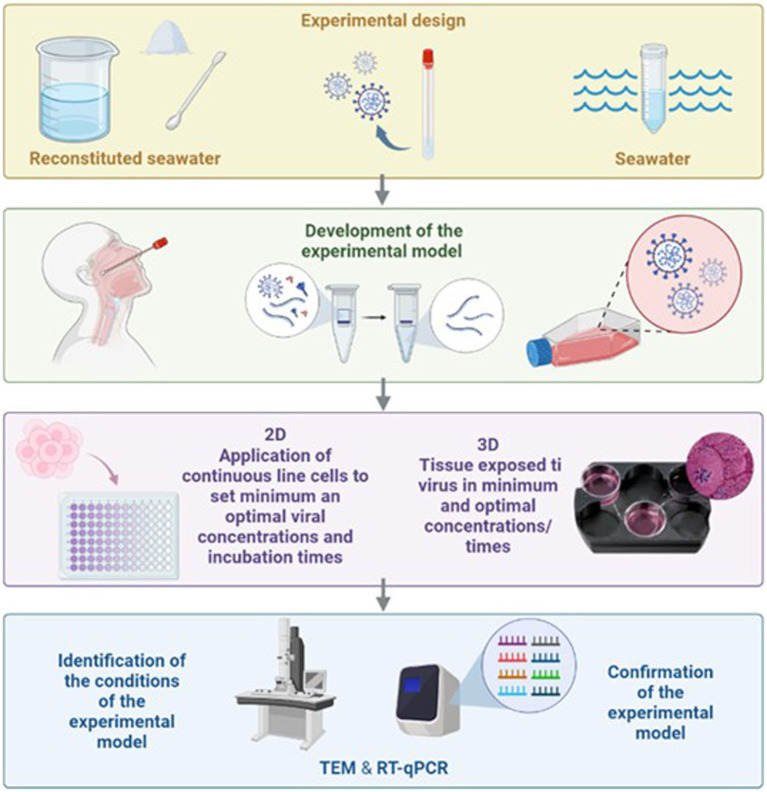

## Introduction

The global COVID-19 pandemic born in Wuhan, China, in late 2019 was driven by the rapid spread of severe acute respiratory syndrome coronavirus 2 (SARS-CoV-2) (1, 2). Various ways of transmission of SARS-CoV-2 have been verified, and others may occur as new public health issues (3). WHO pointed out that swimming pools or crowded beaches feature a risk of the propagation of SARS-CoV-2 through close contact with infected people (4, 5).

A new tool to face this problem is wastewater-based epidemiology (WBE), which is the surveillance of SARS-CoV-2 in wastewater. Analysis of viral contamination in coastal waters stands for a valid public health tool to evaluate infection risk because of the possible ingestion of contaminated water during recreational activities (6, 7). Furthermore, the analysis of viral contamination in coastal waters stands for a valid warning system for monitoring and predicting the circulation of the virus in the population (8–12). WBE can be used to highlight beforehand a possible spread of the virus allowing new epidemic outbreaks to be circumscribed quickly, especially in connection with the territorial health surveillance networks (8). WBE also allows to tracking the circulation of new virus variants in wastewater to secure a public health response (9, 10). Italy has been monitoring the presence of SARS-CoV-2 in wastewater since July 2020 (13). This pilot study has been the premise to set up an Italian structured surveillance network currently referred to as the “SARI project” (SARI protocol rev. 3, 10.5281/zenodo.5758725). The project, coordinated by the Istituto Superiore di Sanità (ISS), has allowed the creation of a network of national territorial facilities (Regional Agencies for Environment Protection, Public Local Health Agencies, Zoo-prophylactic Institutes, Universities, research centers, and integrated water service providers) (14). In March 2021, the SARI project was incorporated into a 24-month Central for Disease Control program by the Ministry of Health, with the participation of 14 Italian Regions. This program was called “Wastewater Epidemiology: Implementation of the Surveillance System for the Early Identification of Pathogens, with Particular Reference to SARS-CoV-2.” Following the publication of the European Commission Recommendation (EU) n. 472 of 17 March 2021 on a common approach to establish a systematic surveillance of SARS-CoV-2 and its variants in wastewaters in the EU, the research activities of the SARI project were converted into a surveillance system that became operational on 1 October 2021 (15).

As a consequence of the health and economic impacts of the COVID-19 pandemic, much of the scientific response has focused on the mechanisms of SARS-CoV-2 entry into host cells, with specific reference to the binding of the viral spike (S) protein to its cell receptor, the angiotensin-converting enzyme 2 (ACE2), and subsequent membrane fusion. Human tissues expressing ACE2 receptors include not only organs belonging to the respiratory system but also to the digestive system including the small intestine and colon (16).

To be pathogenic, viruses must maintain their ability to enter a human cell through the binding of proteins present on its capsid with proteins on the membrane of human cells, acting as an entrance door. Therefore, the mere presence of viral fragments or nucleic acid sequences does not reflect the risk of infection and the consequent risk for public health as demonstrated in airborne SARS-CoV-2 indoor environmental monitoring (17). Thus, to test for the presence of viruses with an infected capacity, it is necessary to use cell lines that can represent the natural target of the virus itself. One limitation of using cell culture as a target is that some viruses are unable to grow or do not induce visible cytopathogenic results (18, 19).

The shedding of infectious SARS-CoV-2 in the feces and urine of COVID-19-infected patients is low or undetectable, despite the detection of viral RNA in these samples and wastewater (20–22). This, together with the rapid inactivation of SARS-CoV-2 in seawater, strongly indicates that the probability of viral transmission through contact with contaminated wastewater is low. The infectivity of SARS-CoV-2 decreases rapidly in seawater, particularly at higher temperatures. The data therefore suggest that seawater contaminated with sewage containing fecal matter from SARS-CoV-2 infected individuals is unlikely to contain high levels of infectious virus due to the rapid inactivation of the virus in these matrices. However, the concentration of the virus in seawater would be significantly diluted compared to, for example, respiratory droplets from an infected person.

In the herein presented study, we proposed an innovative experimental approach to evaluate the infectivity of SARS-CoV-2 present in seawater. We utilized advanced 3D human tissues as hosts for viral infection, which closely mimic the natural target organs of these viruses. Specifically, intestinal tissue model of MatTek (EpiIntestinal™ Ashland, MA) was employed due to its extensive use and well-characterized properties in the pharmaceutical industry. This tissue model exhibits perfect differentiation of enteric cells and retains their native functionality without alteration, offering a more comprehensive alternative to traditional two-dimensional cell cultures, while ensuring reproducibility (23). The EpiIntestinal tissue model expresses crucial receptors, ACE2 and TMPRSS2, which are essential for SARS-CoV-2 entry into target tissues (24). Studies by some research groups demonstrated successful infection of SARS-CoV-2 in ACE2+ mature enterocytes within human small intestinal organoids/enteroids, facilitated by TMPRSS2 and TMPRSS4 to promote virus entry into host cells through SARS-CoV-2 spike binding (25). Accordingly, 3D tissue models represent an advanced approach for accurate testing and prediction of outcomes within a living organism.

The goal of this study was to verify the ability of the SARS-CoV-2 virus to penetrate and overcome the intestinal epithelium. In this regard, the treatment of the 3D epi-intestinal epithelium with SARS-CoV-2 was performed, in both isotonic and controlled salinity conditions. The activity concentrated on defining the environmental reference conditions to evaluate the effects of the permanence of SARS-CoV-2 in salt seawater.

## Materials and methods

### Virus isolation and propagation

Severe acute respiratory syndrome coronavirus 2 was isolated from the nasopharyngeal swabs collected from a pool of 15 patients affected by COVID-19 (10 with Omicron and 5 with Delta variants; Caucasian man of Italian origin whose genomic sequence is available on GenBank; SARS-CoV-2-UNIBS-AP66: ERR4145453). The inoculum of SARS-CoV-2 was carried out on Vero E6 cells (ATCC, Manassas, VA, Number CRL-1586). Vero E6 cells were grown and maintained in modified Eagle Reagent Medium (MEM; Gibco, Waltham, MA, United States) to which 10% heat-inactivated fetal calf serum was added at 37°C in a 5% CO2 humidified atmosphere, following the methodologies reported by Izzotti et al. (17, 26). Given the experience gained in the first tests, in the protocols used for the confirmation analysis of the infection protocol, the amount of fetal calf serum was decreased to 2%.

### Experimental design

The goal of the study was to evaluate to which extent seawater (either natural or reconstructed) decreases SARS-CoV-2 infectivity. Seawater Sars-CoV2-virus was incubated with seawater and thereafter challenged for infectivity in either 2D or 3D cell cultures. 2D cell culture used cells with high expression of ACE2 viral receptor, thus being highly susceptible to virus infection. 3D cell cultures used intestine multilayer human organoids to address the question of whether the ingestion of seawater containing the SARS-CoV-2 virus may represent a risk for viral infection.

Details of experimental conditions used are described as follows: SARS-CoV-2 was incubated with (a) seawater; (b) reconstituted seawater; and (c) phosphate-buffered saline for 2, 10, 30, 60, and 90 min at a 1/15 vol/vol dilution. Subsequently, the maintenance of the infective capacity was evaluated on Vero E6 permissive cells. A sham negative control was used using a nasopharyngeal swabs buffer devoid of SARS-CoV-2 (d). SARS-CoV-2 was incubated with (a) seawater; (b) reconstituted seawater; and (c) phosphate-buffered saline for 2, 10, 30, 60, and 90 min at a 1/15 vol/vol dilution. Subsequently, the maintenance of the infective capacity was evaluated on Vero E6 permissive cells. A sham negative control was used using a nasopharyngeal swabs buffer devoid of SARS-CoV-2 (d).

### SARS-CoV-2 treatment with sea water and reconstituted water

The residual infectivity potential of SARS-CoV-2 was evaluated after treatment with seawater or reconstituted water for five time points. Briefly, 1 mL of concentrated viral inoculum (titer: 7.05 × 10^6^ PFU/mL) was treated with 15 mL of water and incubated at 18°C for 2, 10, 30, 60, and 90 min. At the end of each time point, 1 mL of inoculum was diluted with a complete medium (2% FCS) and used to infect VeroE6 cells for plaque assay in serial dilutions (10^−1^; 10^−2^). SARS-CoV-2 incubated with a physiological solution was used as a positive control of infection.

Seawater was collected from the Ligurian Sea, thus having a hypertonic NaCl concentration of 3.5–3.8%. Reconstituted seawater was prepared by dissolving the compounds listed below (quantities given in grams for 60 L of water) in deionized water: NaF, 0.114 g; SrCl2 6H2O, 0.78 g; H3BO3, 1.20 g; KBr, 4.02 g; KCl, 27.96 g; CaCl2 2H2O, 43.98 g; Na2SO4, 159.60 g; MgCl2 6H2O, 199.80 g; NaHCO3, 7.98 g; and NaCl, 1,659 g. Reconstituted seawater presented a salinity of 3.5% and a pH of 8 achieving 1,000 mOsmol. Reconstituted seawater was sterilized by filtration using a filter having 0.2-μm pores, and physical and chemical parameters were evaluated after this procedure. The artificial water used reflects all the necessary characteristics for the maintenance of aquatic organisms and their cells as documented in the literature. In our department, this ASW (artificial seawater) has been used for a long time to maintain the physiology of intact as well as cells of marine organisms. So that we chose this system because we know it very well and we know how other microorganisms respond. Therefore, we considered salinity, as well as the composition of all salts present, as sufficient parameters, as documented in the study by La Roche et al. The ASW was filtered with a 0.22-μm filter to ensure sterility (27–30). Control phosphate-buffered saline (PBS) solution was composed of 0.01 M phosphate buffer, 0.0027 M potassium chloride, and 0.137 M sodium chloride (pH 7.4) to obtain 300 mOsmol (31). At the end of each contact time, 1 mL of the suspension was diluted in a culture medium for Vero E6 cells and used to infect the cells.

For each treatment [positive control (PBS), seawater, reconstituted seawater, and negative control] and for each treatment time, four experimental replicas were carried out. Accordingly, a total of 80 experimental analyses have been performed. Morphological study was carried out by transmission electron microscopy (TEM).

The Vero E6 cells, treated with SARS-CoV-2 virus as above described, and 3D epi-intestinal tissue were prepared to be visualized by TEM. Cells were washed out in 0.1 M cacodylate buffer and fixed in 0.1 M cacodylate buffer containing 2.5% glutaraldehyde (Electron Microscopy Science, Hatfield, PA, United States), for 1 h at room temperature. The cells were postfixed in 1% osmium tetroxide for 2 h and 1% aqueous uranyl acetate for 1 h. Subsequently, samples were dehydrated through a graded ethanol series and flat embedded in resin (Poly-Bed; Polysciences, Inc., Warrington, PA, United States) for 24 h at 60°C. Ultrathin sections (50 nm) were cut parallel to the substrate and counterstained with 5% uranyl acetate in 50% ethanol. Electron micrographs, as either single snapshots and/or multiple image alignment (MIA), were acquired at Hitachi 7800 120 Kv electron microscope (Hitachi, Tokyo, Japan) equipped with a Megaview 3 digital camera and Radius software (EMSIS, Germany).

### Evaluation of viral infectivity in human intestine 3D model

**Figure 1:**
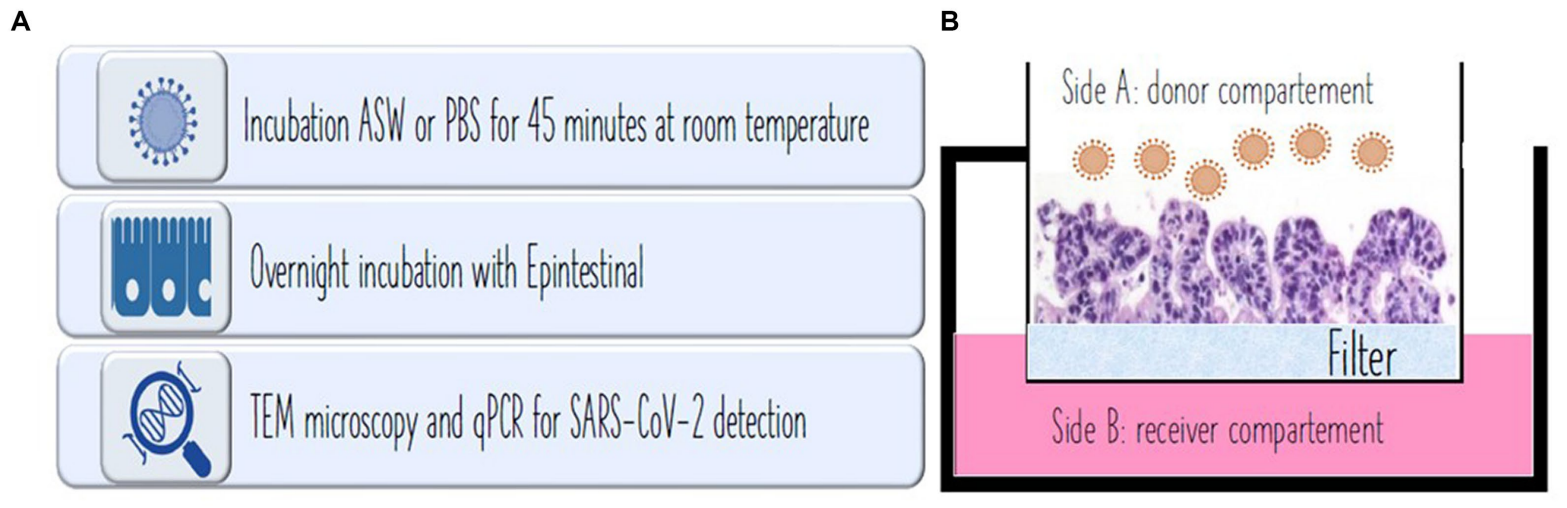
**(A)** Procedure used for the experiment. **(B)** In vivo experiment showing intestinal microvilli at microscope. Side A: donor compartment: virus entrance from the intestinal lumen after water ingestion. Side B: receiver compartment: intestinal capillar representing the system penetration of the virus after intestinal absorption.

The epi-intestinal multi-tissue model was chosen, considered the most interesting with respect to SARS-CoV-2 infection. The 3D tissue is composed of three tissue layers including the intestinal lumen as donor, intestinal epithelium as tissue, and enteric absorption capillaries as the recipient. EpiIntestinal tissues (MatTek, EpiIntestinal™ Ashland, MA, United States) is a 3D human cell-based model that integrates various cell types, including enterocytes, paneth cells, M cells, tuft cells, and intestinal stem cells to form a highly differentiated and polarized epithelium. This advanced model faithfully replicates the complexity and organization of the human intestinal tissue, providing valuable insights into intestinal physiology and pathophysiology. While the primary mode of transmission for the SARS-CoV-2 virus is commonly understood to occur through the respiratory tract, emerging evidence suggests the potential significance of the intestinal tissue as a crucial target organ in the infection and transmission of SARS-CoV-2. Some researchers have revealed that the EpiIntestinal tissue model exhibits strong expression of the ACE2 receptor and TMPRSS2, both crucial for the entry of SARS-CoV-2 into target tissues (24). Clevers et al. (25) have also documented productive SARS-CoV-2 infection in ACE2+ mature enterocytes within human small intestinal organoids/enteroids. This infection is facilitated by TMPRSS2 and TMPRSS4, which promote SARS-CoV-2 spike fusogenic activity, potentially enhancing virus entry into host cells (25, 32). Consequently, the role of small intestinal organotypic and organoids in identifying biomarkers for predicting SARS-CoV-2 infection, transmission, or disease severity warrants the suitability of the model (33). Positive control (PBS), seawater, reconstituted seawater, and negative control (a,b,c,d), prepared as previously described in Experimental design, were incubated with EpiIntestinal tissues overnight at 37°C with 5% CO2 in a humidified atmosphere for virus infection assessment. At the end of the treatment, tissues were immediately fixed (TEM) or frozen (PCR) for further analysis. Figure 1 depicts the steps of the procedure and the compartments composing the epi-intestinal tissues corresponding to the gut lumen and luminal cells (side A: donor compartment) and the intestine capillary (side B: receiver compartment; Figure 1).

Three experimental replicas were made for each one of the three tissue layers and each experimental condition. In addition, untreated 3D tissue was used as a negative control. Accordingly, a total of 39 experimental analyses have been performed.

### Viral titration

Titer determination of infective SARS-CoV-2 virions was performed by plaque assay on VeroE6 cells. VeroE6 was infected with serial dilutions of the treated virus. After 1.5 h of virus absorption, the complete medium with 2% methylcellulose was added. Five days after infection, cells were methanol-fixed, and plaques were stained with crystal violet (0.1%) and counted. Four experimental replicates were performed for each time window.

### Real-time qPCR of viral loads

Severe acute respiratory syndrome coronavirus 2 viral load was determined by amplifying by quantitative real-time PCR (qPCR)-specific gene loci. The amplified genetic material taken into consideration was related to highly conserved segments of the Orf1ab regions and of the gene encoding for the N protein. The Orf1ab gene is linked to the expression of polypeptides, which, following proteolysis, lead to the formation of various proteins with a non-structural function related to the viral life cycle such as proteases and components of the replicase-transcriptase complex (RTC). The N gene refers to the homonymous protein (or nucleocapsid). This is the only SARS-CoV-2 protein capable of binding to the viral genome. Due to this feature, this protein plays a key role in viral RNA synthesis within the new virions.

The methodology chosen for the investigation was the qPCR.

The presence of viral RNA within Vero cells was evaluated by qPCR using the SARS-CoV-2 RT-qPCR Reagent Kit (PerkinElmer, Wathman, MA, United States). The samples were prepared for RNA extraction in an automated robotic station (Janus G3, PerkinElmer, Wathman, MA, United States).

The RNA extraction was carried out using the Chemagic automated station and the related magnetic ball extraction kit (PerkinElmer, Wathman, MA, United States). For each assay, three Taqman qPCR probes were used for (a) housekeeping gene [Ribonuclease P/MRP Subunit P30 (RPP30) used as an internal control]; (b) SARS-CoV-2 Orf1ab viral genes (Vic labeled); and (c) SARS-CoV-2 N viral gene (FAM labeled). The purified RNA was subjected to PCR amplification cycles according to the following parameters: 50°C × 15 min, 95°C × 2 min, 45 cycles at 95°C × 3 s, and 60°C × 30 s. The PCR was performed in a final volume of 20 μL using the LightCycler 480II (Roche).

Comparative quantification was used to detect changes in the genes of interest as compared to a quantity relative to a reference gene, represented by the housekeeping gene Ribonuclease P/MRP Subunit P30 [RPP30] used as an internal control. The approach employed was the Delta Delta Ct (ΔΔCt) method, also known as the Livak method. For each sample, the difference between the Ct values (threshold cycles) of the target gene and of the endogenous control is calculated, obtaining the ∆Ct. Subsequently, subtracting the control condition ∆Ct from the process condition ∆Ct, we calculated the ΔΔCt. The value obtained from this difference was used as the negative exponent of 2 in the 2^−ΔΔCt^ equation. The calculated value represents the difference in the “correct” number of the threshold cycles. The result obtained defines the fold decrease or fold increase of the target genes in the samples, compared to the calibrator sample, normalizing the expression of the reference gene. Results were reported as RNA SARS-CoV-2 copies per mL. The calculation of the infectious viral load reduction, in terms of percentage, was obtained by applying the formula [1–10^(−LR)^] 100.

### Statistical analyses

Statistical analyses were performed using the software R Core Team (2022). R: A language and environment for statistical computing. R Foundation for Statistical Computing, Vienna, Austria. URL: https://www.R-project.org/.

## Results

### Evaluation of viral infectivity

The infectivity reduction results for the two treatments (seawater and reconstituted water) are shown in Tables 1, 2. Values are expressed as logarithmic reduction (LR) compared to the untreated virus. The logarithmic reduction value is the result of the difference between the positive control and the respective number of infecting virions (expressed as plaque-forming units per milliliter).

**Table 1:** Seawater.

Time of incubation	*Log (PFU/mL)*	LR	Infectious viral load reduction (%)
0 min (CTR Positive)	5.672	-	-
2 min	4.788	0.883	86.915
10 min	4.769	0.902	87.482
30 min	4.768	0.904	87.518
60 min	4.770	0.901	87.447
90 min	4.554	1.118	92.376
0 min (CTR Positive)	5.672	-	-
2 min	4.882	0.789	83.759
10 min	4.844	0.828	85.142
30 min	4.777	0.895	87.269
60 min	4.725	0.946	88.688
90 min	4.662	1.009	90.213

Reconstituted seawater. Number of infecting virions expressed as logarithmic viral titer [Log (PFU/mL)], logarithmic reduction (LR), and infectious viral load reduction in terms of percentage after different treatment times (0–90 min).

**Table 2:** The descriptive statistics concerning the results obtained for both seawater and reconstituted seawater at different treatment times (0–90 min).

Treatment	Time (min)	Minimum	1° Quart.	Median	Average	3° Quart.	Max
Control	0	5.5152	5.5339	5.6575	5.6681	5.7879	5.8377
Seawater	2	4.7446	4.7640	4.7926	4.7883	4.8169	4.8235
	10	4.7452	4.7567	4.7679	4.7685	4.7797	4.7931
	30	4.7270	4.7367	4.7671	4.7679	4.7983	4.8102
	60	4.7128	4.7312	4.7726	4.7698	4.8113	4.8213
	90	4.5353	4.5477	4.5538	4.5537	4.5598	4.5717
Reconstituted seawater	2	4.8447	4.8574	4.8840	4.8824	4.9089	4.9169
	10	4.8233	4.8299	4.8445	4.8438	4.8584	4.8629
	30	4.7478	4.7648	4.7772	4.7767	4.7892	4.8048
	60	4.6956	4.7085	4.7250	4.7246	4.7412	4.7529
	90	4.6430	4.6503	4.6622	4.6621	4.6739	4.6808

Each comparison was made by a non-parametric statistical test (Kruskal–Wallis test), which represented the most appropriate choice in relation to the small sample size and the characteristics inherent to the data itself (normality and variance distribution).

The calculation of the reduction in infectious viral load in % terms was instead obtained by applying the formula [1–10^(−LR)^] × 100.

In both conditions tested (seawater and reconstituted seawater), salinity *per se* did not affect cell viability as evaluated by the MTT viability test. Indeed, cell viability was 100.0% in untreated cells, and 99.5 and 99.8% in cells incubated with seawater or reconstituted seawater for 90 min. Accordingly, the results of viral infectivity were not distorted by the effects of possible cellular suffering.

Obtained results provide evidence that seawater dramatically decreases the ability of the SARS-CoV-2 virus to penetrate inside target cells. This effect was fast, being detectable after only 2 min of treatment but further increasing up to 92% of neutralization after 90 min.

In addition, reconstituted seawater dramatically decreased the ability of SARS-CoV-2 virus to penetrate inside target cells. Similarly, in seawater, this effect was fast, being detectable after only 2 min of treatment but further increasing up to 90% of neutralization after 90 min (Table 2).

By comparison, seawater was slightly (2%) more effective than reconstituted seawater in decreasing SARS-CoV-2 infectivity, although this difference was not statistically significant.

Table 3 shows the descriptive statistics concerning the results obtained for both seawater and reconstituted seawater at different treatment times (0–90 min). Each comparison was made by a non-parametric statistical test (Kruskal–Wallis test), which represented the most appropriate choice in relation to the small sample size and the characteristics inherent to the data itself (normality and variance distribution).

**Table 3:** Seawater.

Mathematical model	logLikelihood	AIC	Lack of fit	Residual variance
LogLogistic 3	40.43	−72.86	6.63E−01	0.0012
Weibull 1.3	40.42	−72.84	6.60E−01	0.0012
Cubico	40.98	−71.95	NA	0.0012
Weibull 2.4	40.44	−70.87	3.76E−01	0.0013
LogLogistic 4	40.43	−70.86	3.74E−01	0.0013
Weibull 1.4	40.42	−70.85	3.71E−01	0.0013
Quadratic	35.99	−63.98	NA	0.0019
Linear	27.96	−49.93	NA	0.0040
Weibull 2.3	22.93	−37.86	1.33E−06	0.0070
Cubic	48.40	−86.79	NA	0.0006
Quadratic	47.25	−86.49	NA	0.0006
Weibull 1.4	47.77	−85.54	3.41E−01	0.0006
LogLogistic 4	47.46	−84.92	2.44E−01	0.0006
Linear	45.37	−84.74	NA	0.0007
Weibull 2.4	46.93	−83.87	1.45E−01	0.0007
LogLogistic	40.37	−72.73	2.42E−03	0.0012
Weibull 2.3	40.37	−72.73	2.42E−03	0.0012
Weibull 1.3	40.37	−72.73	2.42E−03	0.0012

Reconstituted seawater. Mathematical models used to describe the number of infecting virions over time after 0–90-min incubation with seawater. For each model, the logLikelihood, AIC, the *p* value relating to the goodness-of-fit test, and the variance of the residuals are reported. The selected model is highlighted in gray.

The intracellular viral loads were significantly different between the two treatments (χ^2^ = 20.82, degrees of freedom = 2, *p* value = 3.01E−05). Dunn’s *post-hoc* test found differences only between seawater and control (*p* value = 1.90E−05), reconstituted seawater and control, but not between seawater and reconstituted seawater (*p* value = 4.70E−04).

**Figure 2:**
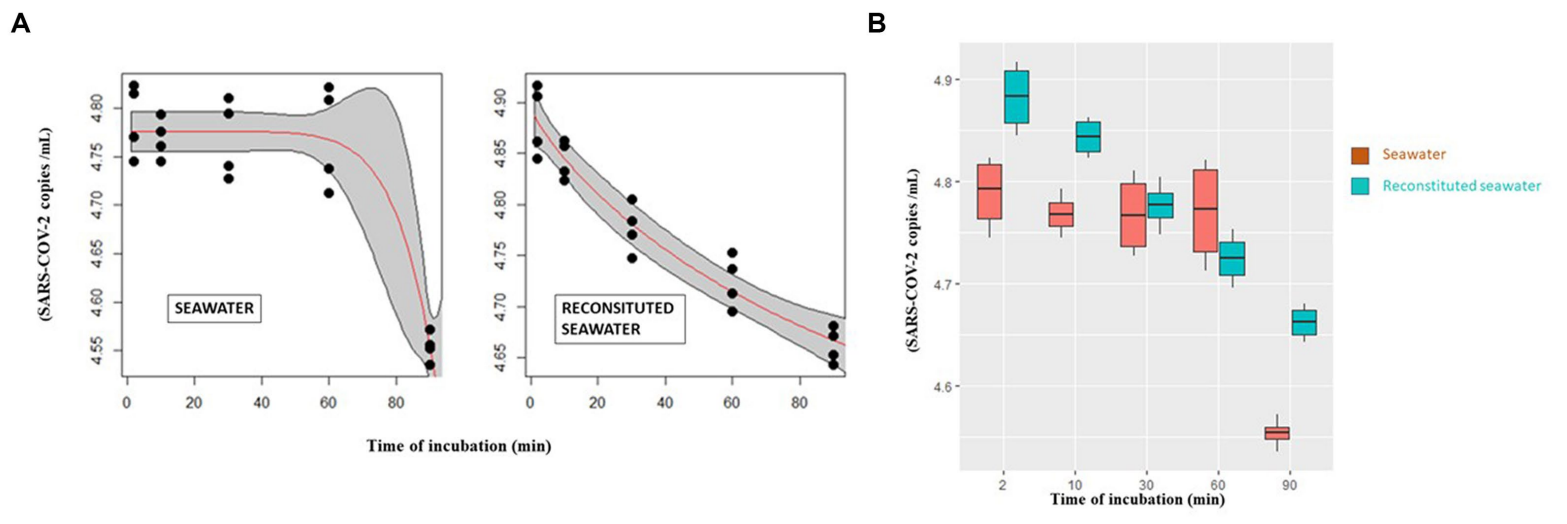
**(A)** Curves describing the abundance of infectious virions after incubation with seawater (left) and reconstituted seawater (right) in relation to time as modeled by Weibull 1.3 and Weibull 1.4 mathematical models, respectively. **(B)** SARS-CoV-2 number of intracellular infecting virions (vertical axis) after different incubation time points (0–90 min) (horizontal axis) with seawater or reconstituted seawater.

As regard seawater, considering the treatment times, differences emerged only when the 2-min series was compared with the 90-min series (*p* value = 2.70E−03; Figure 2A—right) and between 10 and 90 min (*p* value = 2.07E−02, Figure 2A—right).

Linear and non-linear models have been used to describe the reduction of the number of infecting virions over time (Table 4). The choice of the best model was based on the likelihood criterion (Log-likelihood), the Akaike information criterion, and the following outcome of a goodness-of-fit test.

**Table 4:** qPCR of SARS-CoV-2 N gene (copies per mL).

Extended tissue	Treatment	Min.	1° Quart.	Median	Average	3° Quart.	Max	Range	sd
Lumen cells	Isotonic	0.0110	0.0313	0.0385	0.0465	0.0625	0.0884	0.0774	0.0263
Lumen cells	Seawater	0.0179	0.0264	0.0400	0.0496	0.0630	0.1166	0.0987	0.0341
Intestinal epithelium	Isotonic	0.0028	0.0103	0.0272	0.0312	0.0335	0.0825	0.0797	0.0282
Intestinal epithelium	Seawater	0.0063	0.0119	0.0180	0.0169	0.0226	0.0254	0.0191	0.0075
Enteric capillaries	Isotonic	0.0001	0.0005	0.0009	0.0010	0.0010	0.0036	0.0035	0.0011
Enteric capillaries	Seawater	0.0000	0.0005	0.0033	0.0063	0.0091	0.0237	0.0237	0.0082
Lumen cells	Isotonic	0.0039	0.0068	0.0084	0.0105	0.0103	0.0335	0.0296	0.0090
Lumen cells	Seawater	0.0052	0.0071	0.0097	0.0105	0.0129	0.0192	0.0140	0.0046
Intestinal epithelium	Isotonic	0.0001	0.0005	0.0015	0.0019	0.0022	0.0055	0.0054	0.0020
Intestinal epithelium	Seawater	0.0002	0.0007	0.0011	0.0009	0.0013	0.0013	0.0011	0.0004
Enteric capillaries	Isotonic	0.0000	0.0001	0.0002	0.0003	0.0002	0.0008	0.0008	0.0003
Enteric capillaries	Seawater	0.0000	0.0001	0.0014	0.0025	0.0053	0.0063	0.0063	0.0027

Min., Minimum; 1° Quart., First quartile; median, average; 3° Quart., third quartile, maximum, range, and standard deviation (SD) relating to *N* gene for segments analyzed and for treatments.

The data relating to incubation with seawater were modeled using a three-parameter Weibull curve (Weibull 1.3), while a four-parameter Weibull curve (Weibull 1.4) was used to describe the experiments that were performed in reconstituted seawater.

Curves describing the variation of SARS-CoV-2 infectivity after incubation with either seawater or reconstituted seawater are reported in Figure 2B.

Mathematical model analyses indicate that incubation with seawater (Figure 2A, left) sharply decreases the number of virions, especially between 60 and 90 min. Conversely, the curve referring to reconstituted seawater shows a more linear trend (Figure 2A, right).

### Morphological study by transmission electron microscopy

**Figure 3:**
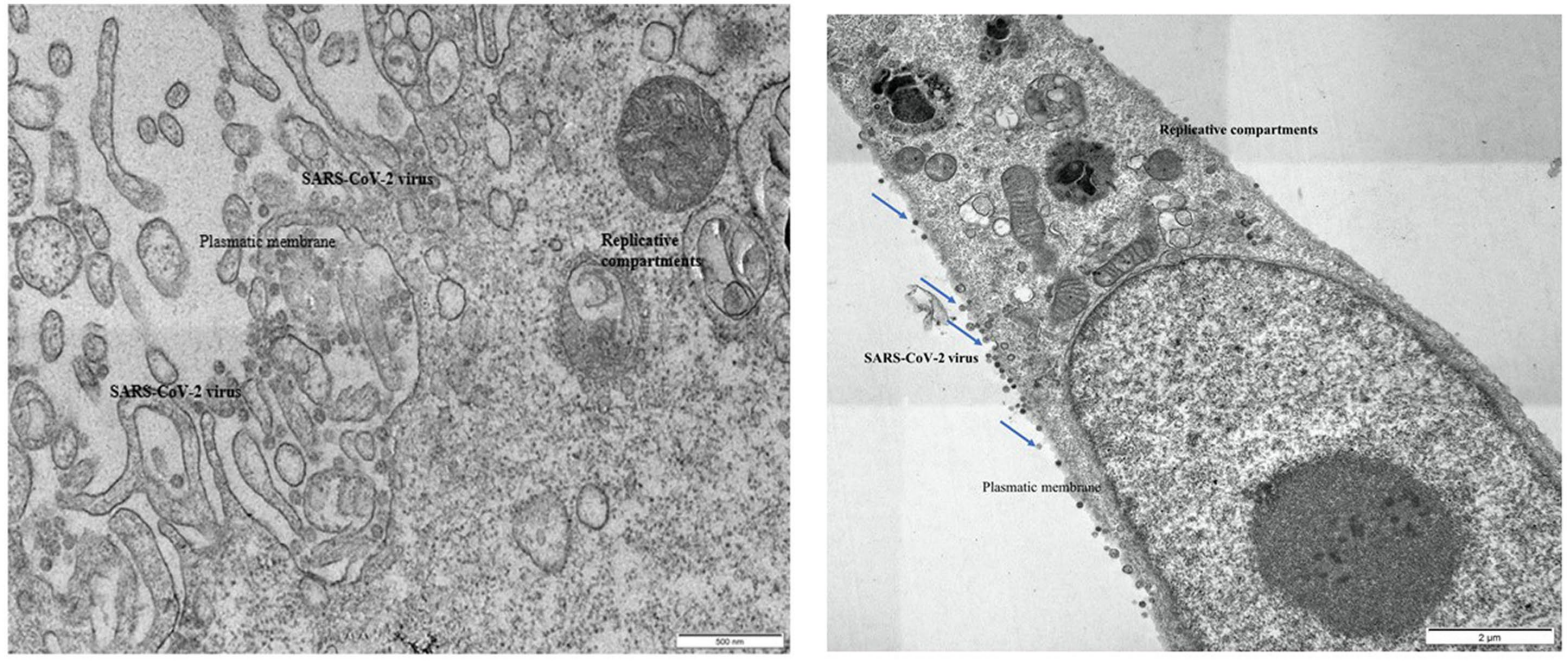
Left: Vero E6 cell. Infected control, untreated with seawater. Right: Vero E6 cell. 2 min in seawater and reconstituted seawater.

**Figure 4:**
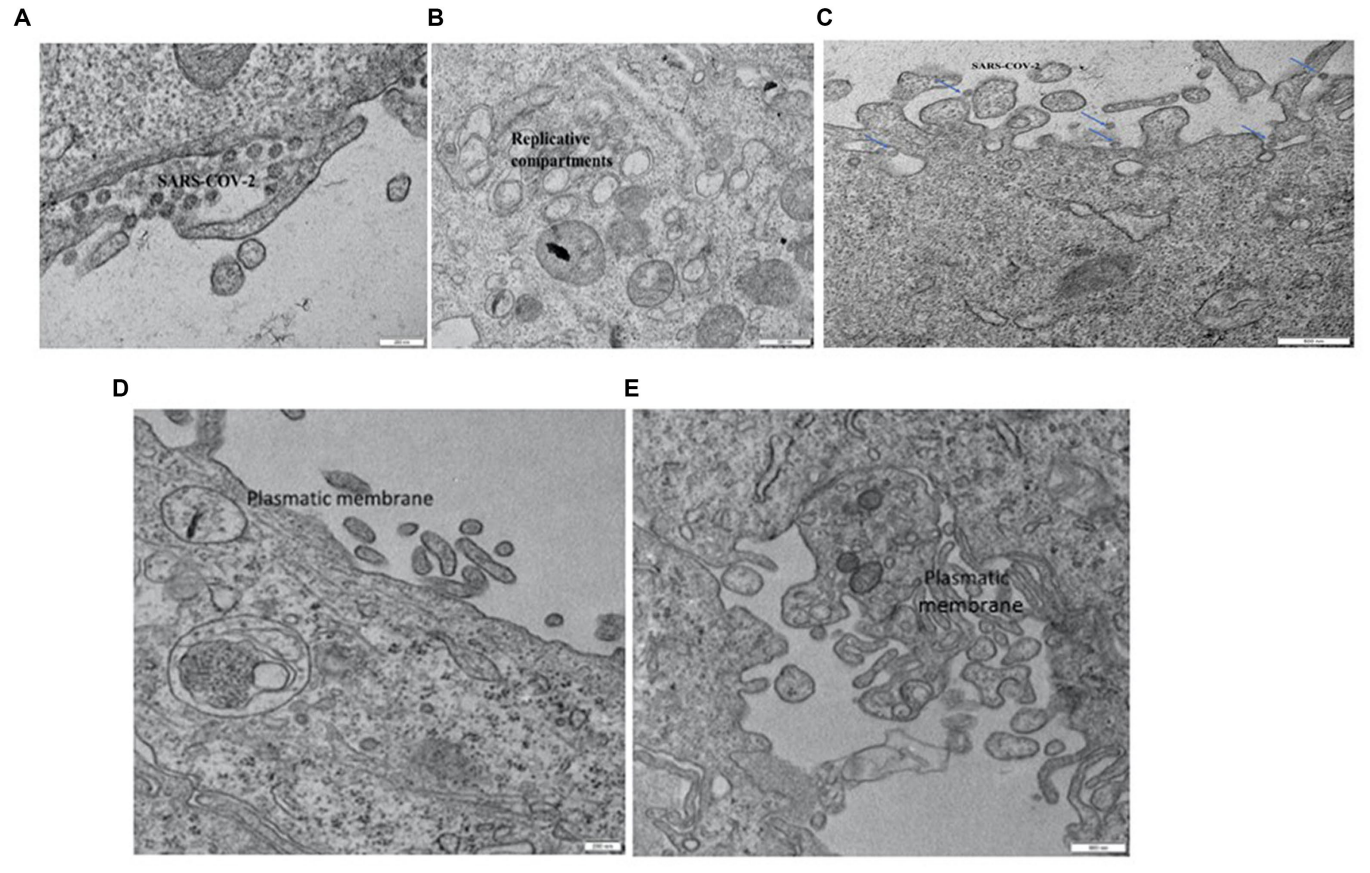
Vero E6 cell. 2 min in seawater **(A)** and reconstituted seawater **(B)**. Vero E6 cell. 10 min in seawater and reconstituted seawater. SARS-CoV-2 components were only occasionally observed on the plasmatic membrane. No replicative compartments were detected **(C)**. Vero E6 cell. 30 min in seawater **(D)** and reconstituted seawater **(E)**. SARS-CoV-2 virus was not detected, and replicative compartments were absent.

**Figure 5:**
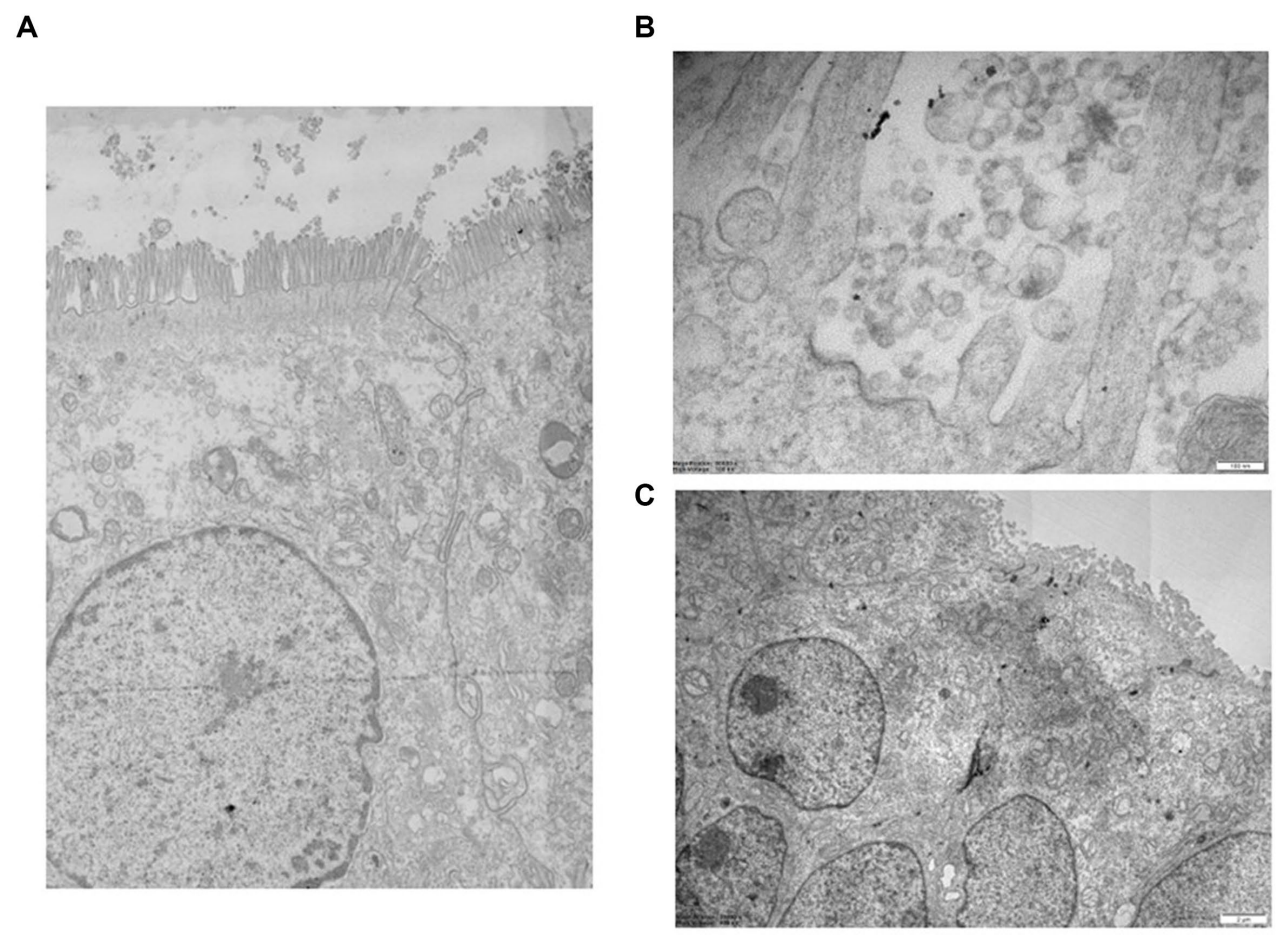
**(A)** Detail of high-resolution MIA collage (5 × 5) of 3D epi-intestinal tissue (MatTek) treated for 24 h with viral swab and visualized by TEM (frontal section). The tissue is well preserved. The intestinal cells show the characteristic “brush border” formed by microvilli on the apical surface. There are no ultrastructural characteristics compatible with an active infection. **(B)** Detail of untreated 3D epi-intestinal tissue (MatTek) (sample 1) visualized by TEM (frontal section, 80.000X). Detail of the “brush border” formed by microvilli on the apical surface with numerous vesicles in the extracellular space. These vesicles do not possess the viral morphological characteristics but can be classified as “extracellular vesicles.” Two invaginations of “clathrin-coated pits,” future endocytic vesicles that are forming, are visible on the cell surface. **(C)** High-resolution MIA collage (5 × 5) of untreated 3D epi-intestinal tissue visualized by TEM (frontal section). The numerous nuclei indicate the presence of an intact epithelium. Numerous mitochondria and the apical surface of the cells decorated with microvilli are observed.

Ultrastructural examination of infected Vero E6 cells revealed the presence of abundant SARS-CoV-2 particles mainly binding extracellularly to the outer cell membrane. Viral presence was observed at the outer plasma membrane also in Vero E6 cells that were incubated with seawater or reconstituted seawater for less than 2 min. Intracellular *bona fide* replication compartments were also observed, but no virions were observed inside them. A significant reduction in the presence of SARS-CoV-2 particles in Vero E6 cells was observed in samples incubated with seawater or reconstituted seawater starting from 10 min and longer incubation time (30, 60, and 90 min). Particularly, after 10 min of incubation, viral particles were only occasionally found extracellularly at the cell surface level, while we did not observe replicative compartments at the intracellular level. These compelling findings, as shown in Figures 3–5, indicate that incubation with seawater for 10 min or more leads to a significant reduction in virus infectivity. These results suggest that prolonged exposure to seawater or reconstituted seawater effectively decreases the ability of the virus to infect target cells, making it a promising approach for mitigating SARS-CoV-2 transmission and enhancing public health safety during recreational activities in seawater environments.

The presence of SARS-CoV-2 in the 3D epi-intestinal model was assessed by ultrastructural analysis after treating the model with viral swabs under various experimental conditions (Figure 5A). The intestinal tissue exhibited excellent ultrastructural preservation under all tested experimental conditions, and cells displayed the characteristic “brush border” formed by microvilli on the apical surface and well-defined nuclei and mitochondria (Figure 5B). No ultrastructural features consistent with active infection were observed (Figure 5C). Numerous vesicles were present in the extracellular space and near the outer cell membrane under all experimental conditions, whether uninfected or infected. However, these vesicles did not possess morphological characteristics compatible with SARS-CoV-2 virions (31, 34). Furthermore, clathrin-coated pits invaginations, which are precursors of endocytic vesicles, were clearly visible on the cell surface. This observation suggests that the intestinal model exhibits active endocytic processes and healthy subcellular compartments without showing signs of infection, indicating the presence of an intact epithelium. Numerous mitochondria and the apical surface of the cells decorated with microvilli are observed.

### Real-time qPCR of viral loads in intestine 3D model

Quantitative real-time PCR detected the intracellular presence of RNA SARS-CoV-2 virus inside the different layers of 3D epi-intestinal (MatTek) tissue infected. Two experimental conditions tested were (a) viral swabs containing SARS-CoV-2 virus mixed with isotonic physiological solution (0.9% NaCl) as control; (b) viral swabs containing SARS-CoV-2 virus mixed with hypertonic seawater (3.8% NaCl).

The purpose of this experiment was to evaluate the effect of seawater salinity in decreasing or increasing SARS-CoV-2 infectivity toward intestinal tissues.

From a quantitative point of view, after 12 h of incubation between SARS-CoV-2-containing samples and intestinal 3D tissue (for both isotonic solution and seawater), the maximum amounts of viral genes were detected in the intestinal lumen cells. Indeed, this is the tissue layer in tight contact with the liquid containing the virus. After 12 h, the viral genes were present in both the intestinal epithelium and the enteric capillaries, although in lesser amounts than those detected in the lumen cells.

**Figure 6:**
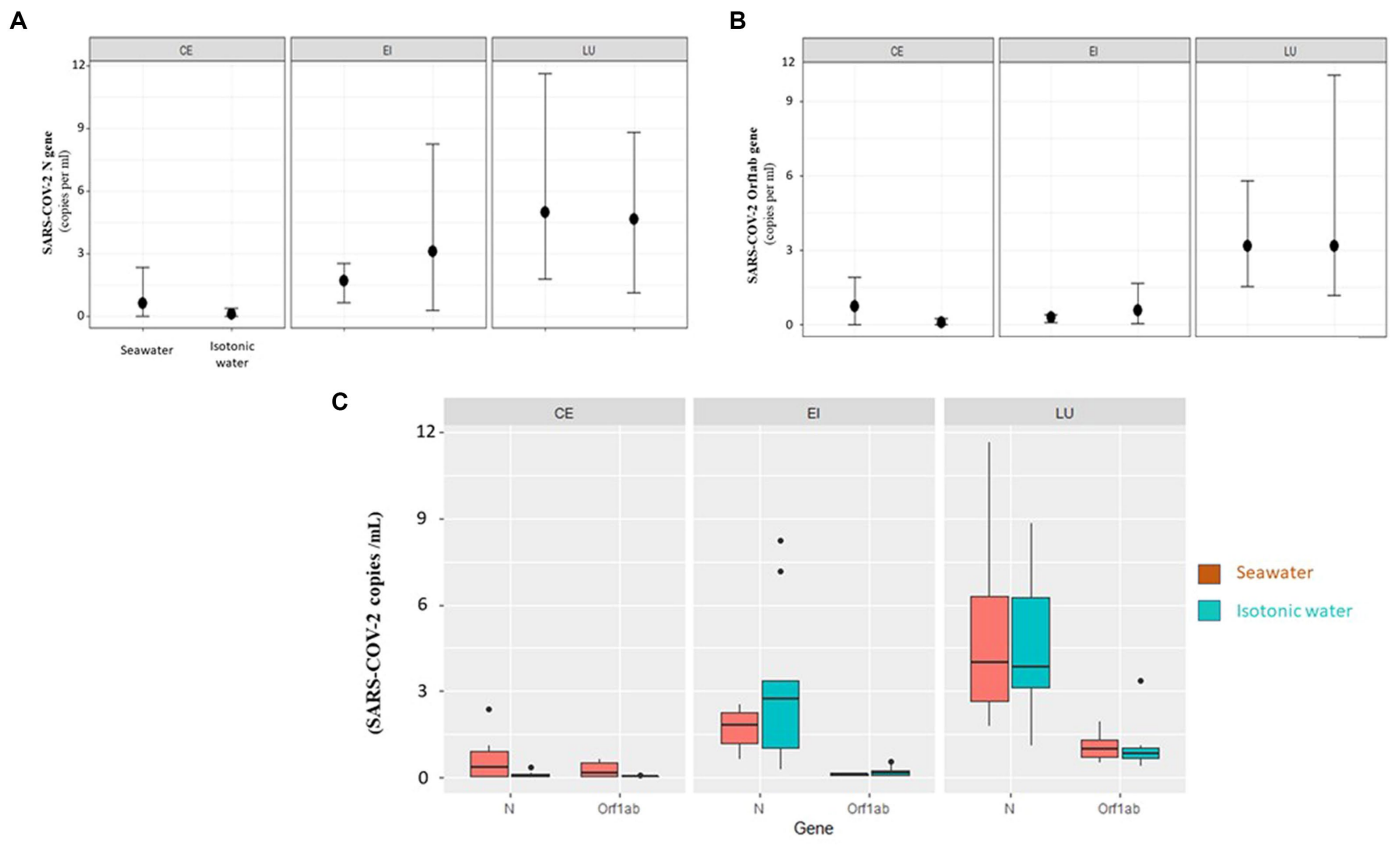
**(A)** Orf1ab gene. Changes of gene expression (average, maximum, and minimum) related to enteric capillaries (CE), intestinal epithelium (EI), and lumen (LU) for the two types of treatment (isotonic and seawater). **(B)** N gene. Changes of gene expression (average, maximum, and minimum) related to enteric capillaries (CE), intestinal epithelium (EI), and lumen (LU) for the two types of treatment (isotonic and seawater). **(C)** Fold value of individual genes (N, Orf1ab) in individual 3D tissue types (CE—enteric capillaries, EI—intestinal epithelium, and LU—lumen) as compared to the medium (seawater and isotonic water).

The variations of viral gene amounts in the different compartments of the 3D intestinal tissues are summarized in Figures 6A,B.

When the three compartments were compared, the lumen (LU) always had the highest values of viral load (*p* value = 5.8E−11, Figure 6C). Viral load progressively decreases in the intestinal epithelium (EI) and is only barely detectable in enteric capillaries (CE). Considering the individual genes, N is the most present in all compartments (*p* value = 5.70E−04, Figure 6C).

## Discussion

Herein presented results provide evidence that seawater can decrease the infectivity of the SARS-CoV-2 virus. As reference experimental models, we decide to use both *in vitro* 2D cell culture (for plaque assay) and 3D organoid culture. For 2D *in vitro* cell culture, Vero E6 cells were selected because their high expression of ACE2 receptors on their membrane makes these cells highly sensitive to SARS. Herein presented results provide evidence that seawater can decrease the infectivity of the SARS-CoV-2 virus. As a reference experimental model, we decide to use both 2D *in vitro* cell culture and 3D organoid culture. For 2D *in vitro* cell culture, Vero E6 cells were selected because their high expression of ACE2 receptors on their membrane makes these cells highly sensitive to SARS-CoV-2 infection, thus providing a very sensitive model to evaluate the modulation of virus infectivity induced by seawater.

For 3D organoid culture, the epi-intestinal model was adopted because reconstructing *in vitro* the complexity of the human intestinal tissues. Indeed, the virus contained in seawater recognized after ingestion as an entry organ the intestinal epithelium.

As a challenge sample, we decided to use the wild virus as directly collected from human COVID-19 affected patients to properly reproduce the on-field situation existing when an affected patient is immersed in marine water.

These experimental models verified that the infectivity of SARS-CoV-2 remarkably decreases following the permanence of the virus in seawater and reconstituted seawater with a time-dependent trend. Although in a different way for both treatments, after a time span of 90 min, the infectivity of the virus is reduced by more than 90%. However, it should be emphasized that for the experimentation in question, the quantity of virus used was deliberately very high to better describe the time-dependent dynamics. Indeed, the whole pool samples collected from COVID-19 patients were directly applied to 2D and 3D cultured cells without any dilution, at variance with the situation occurring under on-field conditions in seawater. Furthermore, it should be considered that when a subject takes a bath in seawater, another important factor contributing to the decrease of virus infectivity is sun radiation. Indeed, Guasp et al. (35) have highlighted a lower incidence of coronavirus in relation to higher sun exposure. Sagripanti and Lytle (36) further illustrated how 90% of SARS-CoV-2 can be inactivated following sunlight exposure from 11 to 34 min. This factor is not considered in our experimental conditions that accordingly overestimate the viral infectivity as compared to the real on-field conditions.

Despite these limits, our results demonstrate that seawater can remarkably decrease virus infectivity after only 10 min of interaction.

Obtained results provided evidence that seawater dramatically decreases the ability of the SARS-CoV-2 virus to penetrate inside target cells. It remains to be established which factors and components of seawater could have contributed to this. A comparison of results obtained with natural or reconstructed seawater may be useful in this regard. Indeed, both water types were able to neutralize SARS-CoV-2 to a similar extent. The virus was not lysed by seawater but still retains its morphology and ability to penetrate inside cells after a very short time incubation with seawater. These findings suggest that the decreased viral infectivity is mainly due to changes induced in viral spike receptors. Indeed, spike protein is highly reactive and electrophilic being composed of amino acids exposing sulfhydryl radicals. Because of this situation, the virus is highly infective but also sensitive to physical–agents such as UV light and oxygen peroxide. The delicate spike protein structure is likely modified by sweater salinity, chemical composition, or osmolality. Indeed, sulfhydryl-rich proteins are highly sensitive to structural and functional modification induced by changes in the ionic strength of the media.

The result of the analysis of viral infectivity on human 3D intestinal tissue models was qualitatively adequate because all intestinal epithelium morphological characteristics were well preserved and visible at TEM. Nevertheless, the morphological analyses have not found the presence of intact viral particles and infection signs in the intestinal tissue cells despite the presence of viral nucleic acid inside intestinal cells as detected by qPCR. This situation may depend on the eclipse phase of the viral infection. Indeed, the ACE2 virus receptor is expressed in the intestinal epithelium (37).

After intracellular penetration, virions were dismounted and no longer detectable by morphological analysis by TEM but only by molecular analyses such as qPCR. This situation is referred to as the “eclipse stage” of viral replication. The virions became again visible only at the end of the intracellular virus replication cycle when the various components of the virus produced by the cell were randomly assembled. This stage of viral replication is indeed referred to as the “assembling stage.” The fact that this stage is not detected at all in infected intestinal epithelium indicates that this tissue may be targeted by viral infection as demonstrated by gastrointestinal symptoms referred to COVID-19-affected patients. However, the intestine, at variance with the respiratory system, does not represent a suitable compartment for SARS-CoV-2 replication and production.

The presence of viral molecular fragments in the absence of detectable whole virions reflects the presence of viral components unable to spread infective virus. This situation is well documented for the SARS-CoV-2 detectable in feces where this virus may be identified by PCR (14). However, the oro-fecal transmission does not represent a mechanism of infectivity for SARS-CoV-2 virus, which is a typically airborne virus for infection spreading.

The virus actively penetrated the intestinal epithelium. After only 12 h of contact, both genes evaluated were present in the intestinal lumen and inside the cells of the intestinal epithelium. However, the virus passed with difficulty through the intestinal epithelium. We found SARS-CoV-2 molecular components in the enteric capillaries in very low quantities, a signal indicating that intestinal tissue does not represent a preferential entry site for SARS-CoV-2 infectivity. Furthermore, the presence of hypertonic saline solution did not increase the sensitivity of the intestinal epithelium to virus penetration.

Our experimental findings are in line with other works dealing with the effects of environmental factors such as sunlight and water salinity on SARS-CoV-2 infectivity.

This virus, like other enveloped viruses, has a low persistence in the environment as well as aquatic matrices (7). Dublineau et al. (38) demonstrated that salinity has a negative effect on enveloped viruses (respiratory viruses) stability as influenza virus. Conversely, Lo et al. (39) showed that non-enveloped viruses (enteric viruses) maintain infectivity in saltwater. Moreover, detergents and proteolytic enzymes present in wastewater may decrease the survival time for enveloped viruses (40). Apart from this, SARS-CoV-2 RNA decay in wastewater is altered by other various factors, such as time outside the host, temperature, and pH (41).

Lee et al. (1) showed a trend like those of our findings when analyzing the SARS-CoV-2 viability and RNA itself, testing high viral titers (10^4^ and 10^5^ PFU/mL) in seawater reporting that SARS-CoV-2 has reduced viability. Only at a concentration higher than 10^5^ PFU/mL (impossible to be found in wastewater and environmental samples undergoing viral dilution), the virus remained viable for 1 day in seawater at 10^5^ PFU/mL while its RNA, more stable than the virus particle itself, persisted longer. Finally, Sala-Colomera et al. (7) used filter-sterilized seawater spiked with infectious SARS-CoV-2 incubated at two different temperatures to estimate the decay rate (time needed to decrease the viral load by 90%). In this study, a lower persistence in seawater than river water was demonstrated with a decay rate of 1.07 and 2.02 day^−1^ at 4 and 20°C, respectively.

The high abundance of SARS-CoV-2 virus in wastewaters poured into seawater has represented a major concern for Public Health during COVID-19 epidemic. The main problem was whether to set up limitations to recreative seawater activities, which would have represented a major problem also for economic reasons, especially in coastal regions of Italy. Results obtained by the herein presented study provide experimental evidence that recreational seawater contaminated with the SARS-CoV-2 virus does not represent a risk to public health because the virus loses the ability to infect sensitive cells when immersed in seawater. Furthermore, it should be highlighted that the high viral loads of the SARS-CoV-2 virus detectable in wastewater are composed of the virus going up from the respiratory system to the laryngopharynx and then being injected into the esophagus and excreted with the feces. These viruses are hot whole virions but are degraded into the intestine. Accordingly, the high viral loads detected by PCR methods in wastewater are composed of RNA fragments belonging to degraded virions.

Overall, the presented data bode well for the quality of bathing water related to possible contaminations by SARS-CoV-2. The results of this study indicate that recreational activities in the seawater environment do not represent a risk factor for COVID-19 infection. The saltwater environment is generally not hospitable for viruses. The high salinity and exposure to sunlight can disrupt the viral envelope, damaging surface viral glycoproteins and making it less infectious. There is currently no evidence that anyone has contracted COVID-19 from swimming in seawater. Recreational activities like swimming, surfing, or diving are not considered to be major risks for COVID-19 transmission.

## Data availability statement

The original contributions presented in the study are included in the article/supplementary material, further inquiries can be directed to the corresponding author.

## Author contributions

EN: Conceptualization, Visualization, Funding acquisition, Writing – review & editing. KC: Formal analysis, Conceptualization, Data curation, Methodology, Writing – review & editing. VG: Formal analysis, Methodology, Writing – review & editing. RR: Formal analysis, Methodology, Writing – review & editing. SR: Formal analysis, Methodology, Writing – review & editing. EG: Formal analysis, Methodology, Writing – review & editing. GN: Formal analysis, Methodology, Writing – review & editing. MG: Formal analysis, Methodology, Writing – review & editing. MT: Methodology, Software, Writing – review & editing. MZ: Methodology, Software, Writing – review & editing. AP: Formal analysis, Visualization, Writing – review & editing. AI: Conceptualization, Validation, Writing – review & editing, Writing – original draft. CN: Writing – review & editing, Writing – original draft.
